# Supplementary material for: Predictors of fatal neurological complications among admitted COVID-19 patients with their implication in outcome: A Case Control study
Source: PLoS One. 2022 Sep 26;17(9):e0274485. doi: 10.1371/journal.pone.0274485 (PMC9512176; doi:10.1371/journal.pone.0274485)
Supplement: Attachment — Submitted filename: PONE-D-22-05696_reviewer.pdf [file pone.0274485.s002.pdf]

PLOS ONE

Predictors of Fatal Neurological Complications among Admitted COVID-19 Patients with their Implication in Outcome: A Case Control Study

--Manuscript Draft--

**Manuscript Number:**	PONE-D-22-05696
**Article Type:**	Research Article
**Full Title:**	Predictors of Fatal Neurological Complications among Admitted COVID-19 Patients with their Implication in Outcome: A Case Control Study
**Short Title:**	predictors and outcome of fatal neurological complicatins among admitted COVID-19 patients
**Corresponding Author:**	Javaria Aslam, MBBS, FCPS Medicine<br>Quaid-e-Azam Medical College<br>Bahawalpur, Punjab PAKISTAN
**Keywords:**	Fatal neurological complications; COVID-19; Predictors; Outcome
**Abstract:**	Abstract<br>Background<br>COVID-19 is known to be associated to potentially fatal neurological complications.<br>Aims<br>To determine the risk factors for developing fatal neurological complications and their outcome in hospitalized COVID-19 patients.<br>Material and Methods<br>Case control study based on hospitalized patients was conducted from July 15th 2021 to December 15th 2021. Cases and controls were COVID-19 confirmed patients with and without severe neurological manifestations. Age, comorbid conditions, vaccination status, Blood Sugar Random (BSR), D-dimers levels, anticoagulation type and dosage were taken as predictors for neurological squeale. Cases were further analyzed for primary outcome of 28 day mortality using the same factors as predictor. Chi square test and regression model were built to calculate OR with 95%CI.<br>Results<br>Total 383 analyzed populations comprised 95 cases and 288 controls. Median age was 56, IQR (27,110), 50.5% females, 67(70.5%) developed CVA, 21(22.1%) Encephalitis and 7(7.4%) GBS among cases. Twenty two (23.2%) from cases Vs. 39(13.5%) controls were from age group ≥71 yrs. OR 1.9, 95%CI (1.1, 3.5) and p<.05. Thirty six (37.9%) cases vs. 69(24%) had DM, OR 1.9, 95%CI (1.2, 3.1), p <.05. Nine (9.5%) cases Vs. 70(18.3%) were vaccinated, OR 0.33, 95% CI (0.16, 0.68), p<.01. Nineteen (20%) cases vs. 0 had admission hyperglycemia (BSR 351-600 mg/dl), OR 5.1, 95%CI (2.7, 9.4), p<.01. Thirty nine (41%) cases Vs. 34(11.8%) had D-dimers level 5,000-10,000 OR 5.20, 95%CI (3.02, 8.9). Eighty three (86.3%) cases Vs. 130(45.1%) had disease progression to death, OR 7.66, 95%CI (4.08, 14.4), RR 5.1 95%CI (2.93, 8.81) and p<.001. Unvaccinated patients with neurological complications had increased risk of death, OR 6.84, 95%CI (1.55, 30.22), P<.05. Death outcome was more among patients with CVA, OR 7.46, 95%CI (2.06, 26.96), p<.01.<br>Conclusion<br>Old age, presence of Diabetes Mellitus, unvaccinated status of patients, high BSR and D-dimers, and prophylactic dose anticoagulation were found to be increased risk factors for developing serious neurological complication whereas they had on impact on death outcome among cases. Cerebrovascular Accidents were the commonest complication with increased mortality followed by encephalitis.
**Order of Authors:**	Javaria Aslam, MBBS; FCPS Medicine
	Shoaib Luqman, MBBS; FCPS Neurology
	Sadaf Nazly, MBBS
	Alina Saeed, MBBS
	Muhammad Sohail Tariq, MBBS; FCPS Medicine
	Irrum Aneela, MBBS; MRCP

Powered by Editorial Manager® and ProduXion Manager® from Aries Systems Corporation

Additional Information:	
Question	Response
**Financial Disclosure**<br><br>Enter a financial disclosure statement that describes the sources of funding for the work included in this submission. Review the submission guidelines for detailed requirements. View published research articles from *PLOS ONE* for specific examples.<br><br>This statement is required for submission and **will appear in the published article** if the submission is accepted. Please make sure it is accurate.<br><br>**Unfunded studies**<br>Enter: *The author(s) received no specific funding for this work.*<br><br>**Funded studies**<br>Enter a statement with the following details:<br>• Initials of the authors who received each award<br>• Grant numbers awarded to each author<br>• The full name of each funder<br>• URL of each funder website<br>• Did the sponsors or funders play any role in the study design, data collection and analysis, decision to publish, or preparation of the manuscript?<br>• **NO** - Include this sentence at the end of your statement: *The funders had no role in study design, data collection and analysis, decision to publish, or preparation of the manuscript.*<br>• **YES** - Specify the role(s) played.<br><br>* typeset	The author(s) received no specific funding for this work.
**Competing Interests**<br><br>Use the instructions below to enter a competing interest statement for this submission. On behalf of all authors, disclose any competing interests that could be perceived to bias this work—acknowledging all financial support and any other relevant financial or non-financial competing interests.	The authors have declared that no competing interests exist.

This statement is required for submission and **will appear in the published article** if the submission is accepted. Please make sure it is accurate and that any funding sources listed in your Funding Information later in the submission form are also declared in your Financial Disclosure statement.<br><br>View published research articles from *PLOS ONE* for specific examples.<br><br>**NO authors have competing interests**<br><br>Enter: *The authors have declared that no competing interests exist.*<br><br>**Authors with competing interests**<br><br>Enter competing interest details beginning with this statement:<br><br>*I have read the journal's policy and the authors of this manuscript have the following competing interests: [insert competing interests here]*<br><br>* typeset	
**Ethics Statement**<br><br>Enter an ethics statement for this submission. This statement is required if the study involved:<br><br>• Human participants<br>• Human specimens or tissue<br>• Vertebrate animals or cephalopods<br>• Vertebrate embryos or tissues<br>• Field research<br><br>Write "N/A" if the submission does not require an ethics statement.<br><br>General guidance is provided below. Consult the submission guidelines for detailed instructions. **Make sure that all information entered here is included in the**	institutional Ethical Review Board, Qauid e Azam Medical College Bahawalpur.<br>no. 1283/DME/QAMC/Bahawalpur, Pakistan.

**Methods section of the manuscript.**<br><br>**Format for specific study types**<br><br>**Human Subject Research (involving human participants and/or tissue)**<br>• Give the name of the institutional review board or ethics committee that approved the study<br>• Include the approval number and/or a statement indicating approval of this research<br>• Indicate the form of consent obtained (written/oral) or the reason that consent was not obtained (e.g. the data were analyzed anonymously)<br><br>**Animal Research (involving vertebrate animals, embryos or tissues)**<br>• Provide the name of the Institutional Animal Care and Use Committee (IACUC) or other relevant ethics board that reviewed the study protocol, and indicate whether they approved this research or granted a formal waiver of ethical approval<br>• Include an approval number if one was obtained<br>• If the study involved *non-human primates*, add *additional details* about animal welfare and steps taken to ameliorate suffering<br>• If anesthesia, euthanasia, or any kind of animal sacrifice is part of the study, include briefly which substances and/or methods were applied<br><br>**Field Research**<br><br>Include the following details if this study involves the collection of plant, animal, or other materials from a natural setting:<br>• Field permit number<br>• Name of the institution or relevant body that granted permission	
**Data Availability**<br><br>Authors are required to make all data underlying the findings described fully available, without restriction, and from the time of publication. PLOS allows rare exceptions to address legal and ethical concerns. See the PLOS Data Policy and FAQ for detailed information.	Yes - all data are fully available without restriction

A Data Availability Statement describing where the data can be found is required at submission. Your answers to this question constitute the Data Availability Statement and **will be published in the article**, if accepted.<br><br>**Important:** Stating 'data available on request from the author' is not sufficient. If your data are only available upon request, select 'No' for the first question and explain your exceptional situation in the text box.<br><br>Do the authors confirm that all data underlying the findings described in their manuscript are fully available without restriction?	
**Describe where the data may be found in full sentences. If you are copying our sample text, replace any instances of XXX with the appropriate details.**<br><br>• If the data are **held or will be held in a public repository**, include URLs, accession numbers or DOIs. If this information will only be available after acceptance, indicate this by ticking the box below. For example: *All XXX files are available from the XXX database (accession number(s) XXX, XXX.*).<br>• If the data are all contained **within the manuscript and/or Supporting Information files**, enter the following: *All relevant data are within the manuscript and its Supporting Information files.*<br>• If neither of these applies but you are able to provide **details of access elsewhere**, with or without limitations, please do so. For example:<br><br>*Data cannot be shared publicly because of [XXX]. Data are available from the XXX Institutional Data Access / Ethics Committee (contact via XXX) for researchers who meet the criteria for access to confidential data.*<br><br>*The data underlying the results*	Data of study was manually taken from patients electronic record and charts of patients, it was taken into excel files and will be made available before or after acceptance as required by journal

presented in the study are available from (include the name of the third party and contact information or URL).<br>• This text is appropriate if the data are owned by a third party and authors do not have permission to share the data.<br><br>* typeset	
Additional data availability information:	Tick here if your circumstances are not covered by the questions above and you need the journal’s help to make your data available.

Powered by Editorial Manager® and ProduXion Manager® from Aries Systems Corporation

Manuscript Click here to access/download;Manuscript;Manuscript.docx

Predictors of Fatal Neurological Complications among Admitted COVID-19 Patients with Their Implication in Outcome: A Case Control Study

Javaria Aslam[1], Shoaib Luqman[2], Sadaf Nazly[1], Alina Saeed[1], Muhammad Sohail Traiq[1], Irrum Aneela[3]

[1]Department of Medicine, Sir, Sadiq Abbasi Hospital, Qauid e Azam Medical College, Bahawalpur, Pakistan.

[2]Department of Neurology, Qauid e Azam Medical College Bahawalpur Pakistan

[3]Stroke Medicine Departmet, Fairfield General Hospital, National Health System, Bury, United Kingdom

Corresponding author:

[1]Javaria Aslam

Javaslam50@gmail.com

Abstract

Background

COVID-19 is known to be associated ~~to~~ potentially fatal neurological complications

Aims

To determine the risk factors for developing fatal neurological complications and their outcome in hospitalized COVID-19 patients.

Material and Methods

Case control study based on hospitalized patients was conducted from July 15th 2021 to December 15th 2021. Cases and controls were COVID-19 confirmed patients with and without severe neurological manifestations. Age, comorbid conditions, vaccination status, Blood Sugar Random (BSR), D-dimers levels, anticoagulation type and dosage were taken as predictors for neurological squeale. Cases were further analyzed for primary outcome of 28 day mortality using the same factors as predictor. Chi square test and regression model were built to calculate OR with 95%CI.

Results

Total 383 analyzed populations comprised 95 cases and 288 controls. Median age was 56, IQR (27,110), 50.5% females, 67(70.5%) developed CVA, 21(22.1%) Encephalitis and 7(7.4%) GBS among cases. Twenty two (23.2%) from cases Vs. 39(13.5%) controls were from age group ≥71 yrs. OR 1.9, 95%CI (1.1, 3.5) and p<.05. Thirty six (37.9%) cases vs. 69(24%) had DM, OR 1.9, 95%CI (1.2, 3.1), p <.05. Nine (9.5%) cases Vs. 70(18.3%) were vaccinated, OR 0.33, 95% CI (0.16, 0.68), p<.01. Nineteen (20%) cases vs. 0 had admission hyperglycemia (BSR 351-600 mg/dl), OR 5.1, 95%CI (2.7, 9.4), p<.01. Thirty nine (41%) cases Vs. 34(11.8%) had D-dimers level 5,000-10,000 OR 5.20, 95%CI (3.02, 8.9). Eighty three (86.3%) cases Vs. 130(45.1%) had disease progression to death, OR 7.66, 95%CI (4.08, 14.4), RR 5.1 95%CI (2.93, 8.81) and p<.001. Unvaccinated patients with neurological complications had increased risk of death, OR 6.84, 95%CI (1.55, 30.22), P<.05. Death outcome was more among patients with CVA, OR 7.46, 95%CI (2.06, 26.96), p<.01.

Conclusion

Old age, presence of Diabetes Mellitus, unvaccinated status of patients, high BSR and D-dimers, and prophylactic dose anticoagulation were found to be increased risk factors for developing

serious neurological complication whereas they had on impact on death outcome among cases. Cerebrovascular Accidents were the commonest complication with increased mortality followed by encephalitis.

Key Words

Serious, Neurological Complications, Risk Factors, Death Outcome.

Introduction

Although COVID-19 is typified by respiratory symptoms and complications, neurological presentations of disease must not be underestimated. At least one subjective neurological symptom has been reported in nearly 36 to 85% of COVID-19 patients, highlighting the importance of the disease's neurological repercussions.(1)(2) Initially, headache, gustatory and olfactory dysfunctions and confusion were the most common general non-specific neurological symptoms reported by COVID-19 patients while major neurological problems such as ischemic and hemorrhagic stroke, cerebral (sinus) venous thrombosis, Encephalitis, Guillain–Barré syndrome and posterior reversible encephalopathy syndrome (PRES) had also been observed with severe cases.(3)(4) With the introduction of variants of concern (VOC), such as the delta variant (B.1.167.2) of SARS-COV-2, the disease's severity and complications had been escalated.(5) Patients with severe COVID-19 infection had a higher rate of neurological complications than those with moderate infection. Moreover, neurological complications may have poor impact on outcome of COVID-19 patients. (6)(7)

Neuropathology is caused by a combination of immunological and hypoxia-induced damage superimposed by coagulation abnormalities(8). ACE 2 receptors being the principal binding sites for SARS-COV-2, are found in neurons, oligodendrocytes, astrocytes, substantia nigra, posterior cortex, brainstem and olfactory bulb. As a result, SARS-CoV-2 has the ability to infect neurons and glial cells throughout the brain. (9) (8) Blood-brain barrier(BBB) is broken by elevated cytokine levels, chemokine, and free radicals linked to activation of the immune system. Triggered inflammatory cascade can cause reactive astrogliosis .(10) Once the virus has crossed the BBB and entered the CNS, it will be difficult to eliminate it since the nervous system lacks substantial histocompatibility antigens and the immune response will be confined to cytotoxic T cells. The patient finally develops acute encephalitis, viral toxic encephalopathy, or acute

cerebrovascular accidents (CVAs) (8)(10). Infectious toxic encephalopathy (ITE) is a reversible brain dysfunction syndrome marked by cerebral edema caused by systemic toxemia and hypoxia, which can result in delirium and coma. In COVID-19 patients, virus-induced cytokine storm and coagulation abnormalities increase the likelihood of acute CVA.(11)

There are several clinical, laboratory and radiological investigations that can predict the severity of disease and outcome in COVID-19 patients assigned to respiratory complications, (12) but there are hardly any relevant predictors for neurological sequelae. So, we designed an observational study at COVID-19 High Dependency Unit of tertiary care hospital to investigate possible predicting factors that may put COVID-19 patients at risk of developing serious neurological complications, as well as to observe any impact of complications and predictors on patients' outcome.

Material and Methods

A Case Control study approved by Institutional Review Board of Qauid e Azam Medical College Bahawalpur was carried out between July 15th, 2021 and December 15th 2021, delta period of SARS-COV-2 in Pakistan, to find out risk factors for fatal neurological complications in admitted COVID-19 patients. Study comprised adults aged 18 years and above for potential eligibility through daily review of hospital admissions and electronic record of COVID-19 High Dependency Unit and Intensive Care Units, at Sir Sadiq Abbasi Hospital, Bahawalpur, a tertiary care hospital that receives referrals of COVID-19 confirmed patients from all primary, secondary, and tertiary care hospitals of division. An informed consent was taken from patients or their escorts. The STROBE (Strengthening the Reporting of Observational Studies in Epidemiology) reporting guideline was followed in this study.

Cases were Patients with a clinical syndrome consistent with acute COVID-19 and a positive molecular test (RT-PCR) for SARS-CoV-2 or suggested HRCT who were hospitalized within 10 days of symptom onset and developed an acute onset severe neurological complication at admission or within 28 days of hospital stay. Neurological manifestations were defined as recent onset focal neurological deficit or altered sensorium gained after having symptoms of COVID-19 or laboratory confirmation of it. In addition to a full neurological evaluation by a neurologist, neuroimaging such as computed tomography, MRI brain, CSF analysis, and Nerve Conduction

Studies were performed as required. The study excluded participants having a history of previous neurological illnesses that left them with residual weakness. Since, the study was focused on serious neurological manifestations as a complication of COVID-19, so mild neurological symptoms e.g. loss of taste and smell, headache and facial nerve palsy were excluded. Controls were gender and duration matched at a 3:1 ratio from lists of eligible COVID-19 confirmed participants who had been sick enough to get admitted to the hospital within two weeks of the case' enrollment. Matching for admission date was a strategy to eliminate the possibility of bias due to time as the disease severity was reported to be significantly ferocious during the delta period of SARS-COV-2.

Demographic characteristics including age and gender were noted, on basis of age were divided into three main age groups. Co morbid conditions and their number in each participant were also noted, mainly focusing on uncontrolled comorbid factor for severe COVID-19. Blood Sugar Random in mg/dl was also noted at time of hospitalization; however, it altered subsequently due to hospital and disease-related stress, as well as intravenous steroid. D-dimers were noted within 24 hours of admission to hospital before start of anticoagulation. The D-dimer result was expressed in ng/ml FEU (Fibrinogen equivalent unit). To categorize COVID-19 severity, we used a customized version of the World Health Organization COVID-19 Clinical Progression Scale, which goes from infection naïve (level 0) to infected albeit asymptomatic (level1) to death (level 9). To determine severity, the patient's highest ordinal level within the first 28 days of hospitalization was employed. The highest severity level observed could range from level 4 to level9, including hospitalized with no oxygen support (level4), with standard oxygen therapy (level5), with high-flow nasal cannula or noninvasive ventilator support (level6), with mechanical ventilation (level7), or with mechanical ventilation and additional organ assistance (hemodynamic support, extracorporeal membrane oxygenation, or new Renal Replacement Therapy (level 9).According to scale level 4 were categorized as moderate disease, level 5 as severe disease and level 6,7 and 8 as critical illness. Patients' vaccination status was determined by entering their National Identity numbers, which were obtained from their hospital electronic records, into a national database of vaccination program, along with the dates of their first and second doses of vaccination, and categorizing them as fully vaccinated or unvaccinated. Anticoagulation type given during hospital stay or before admission to hospital was also noted in the form of oral anticoagulants, Low Molecular Weight Heparin (LMWH) and unfractionated

heparin. Anticoagulation dose with reference to thromboprophylaxis (Enoxaparin 40mg daily, 60mg daily if body weight >100 kg, Rivaroxaban 10mg daily) and therapeutic (Enoxaparin 1mg/Kg body weight twice daily, Rivaroxaban 15 or 20 mg daily) were also noted. In statistical analysis, median with IQR were taken for continuous variables and frequencies and percentages for categorical variables. The adjusted and unadjusted odds ratios (ORs) with their 95%CI for exposure to predictable variables and outcome were compared. Chi Square test of independence and logistic regression model were created to calculate OR and significance values for dichotomous and multilevel dependent variables wherever applicable, keeping p value <.05 to be statistically significant. In subgroup analysis, cases with neurological complications were assessed for predictive variables and outcome in terms of illness progression to death, survival leading to discharge from the hospital with residual weakness, or complete remission of neurological manifestations. OR with 95% CI for death outcome were calculated with help of Chi Square test and regression model, keeping p value <.05 as statistically significant. Odds Ratio for whom a 95% Confidence Interval crossing the null is considered insignificant. SPSS version 20 was for data analysis.

Results

During study period, total 105 cases were enrolled, 10 patients were excluded, and the major cause of exclusion was previous history of neurological illness with residual weakness and undiagnosed neurological manifestation. Total 383 analyzed populations comprised 95 cases and 288 gender and duration matched controls. Median age was 56, IQR (27,110) and 56, IQR (21,99) among cases and controls respectively. Forty eight (50.5%) were female among cases. Among cases 67(70.5%) patients developed Cerebral Vascular Events, seven (7.4%) patients had GBS confirmed by Nerve Conduction Study and 21(22.1%) patients were diagnosed to have Encephalitis. Among patients with CVA, 55(82%) had Ischemic infarct of arterial territory on Computed Tomography, six (9%) had hemorrhagic infarct and six (9%) patients had cerebral venous thrombotic events including three cases with cavernous venous sinus thrombosis. Frequency and percentages for categorical variables and median with IQR for continuous variables among cases and controls are given in table.1.

Table.1. Characteristics of case patients (with neurological complications) and control patients (without neurological complications)

Characteristics	No. (%) Cases (n=95)	Controls (n= 288)
Age Median, IQR	56(27,110)	56(25,99)
Age groups		
25-45 yrs.	16(16.8)	67(23.3)
46-70 yrs.	57(60)	182(63.2)
≥71 yrs.	22(23.2)	39(13.5)
No. of comorbid conditions Median, IQR	1(0,4)	1(0,4)
Comorbid conditions		
No risk factor	24(25.3)	87(30.2)
DM	36(37.9)	69(24)
Cardiovascular diseases	27(28.4)	108(37.5)
Pulmonary disease	1(1.1)	11(3.8)
Obesity	0	1(0.3)
Old Age	7(7.4)	12(4.2)
Vaccination status		
Vaccinated	9(9.5)	70(18.3)
Unvaccinated	86(90.5)	218(75.7)
BSR(mg/dl) Median, IQR	218(80,578)	174(98,340)
<200	37(38.9)	206(71.5)
201-350	30(31.6)	82(28.5)
351-600	19(20)	0
D-dimers(ng/ml)	5900(280,13,000)	2100(320,7900)
≤500	9(9.5)	12(4.2)
501-5000	28(29.5)	225(78.1)
5000-10,000	39(41)	34(11.8)
>10,000	19(20)	17(5.9)

Twenty two (23.2%) cases Vs. 39(13.5%) were from age group ≥71 yrs. with OR 1.9, 95%CI (1.1, 3.5) and $p<.05$. Thirty six (37.9%) cases Vs. 69(24%) were having Diabetes Mellitus as a

risk factor for severe disease with OR 1.9, 95%CI (1.2, 3.1), p <.05. Nine (9.5%) cases Vs. 70(18.3%) were vaccinated, OR 0.33, 95% CI (0.16, 0.68), p<.01. Estimated vaccine effectiveness against development of neurological complications is (1-0.33) ×100= 66.9%. Nineteen (20%) cases vs. 0 controls had admission hyperglycemia of 351-600 mg/dl even if they were not known diabetic before start of illness, OR 5.1, 95%CI(2.7, 9.4), p<.01. Thirty nine (41%) cases Vs. 34(11.8%) had D-dimers level 5,000-10,000 OR 5.20, 95%CI (3.02, 8.9). So, patients who had higher D-dimers >5,000 had 420% more chances to get neurological complications than those who had less high levels of D-dimers.

Eighteen (18.9%) patients among cases did not have hypoxemia at admission to hospital Vs. 11(3.8%) controls with OR .17, 95% CI (.07, .37) P<.001. Nineteen (20%) cases Vs. 11(3.8%) had moderate disease, 30(31.6%) Vs. 117(40.6%) had severe disease and 46(48.4%) Vs. 160(55.6%) had critical illness. Eighty three (86.3%) cases Vs. 130(45.1%) had disease progression to death or shifting to invasive ventilator support, OR 7.66, 95%CI (4.08, 14.4), RR 5.1 95%CI (2.93, 8.81) and p<.001. So, patients who had neurological complications had 660% more chances to die with COVID-19 once they need admission for their illness. Association between predictor variables and cases and controls are given in Figure.1 with respect to their OR 95%CI where effect sizes are shown in forest plot.

Figure 1.. Association between predictor variables and development of neurological complications

Characteristics	Cases n (%)	Controls n (%)	Absolute difference 95%CI	Adjusted OR 95%CI
Age groups, yrs				
25-45	16(16.8)	67(23.3)	-6.5(-3.4 to 14.6)	.126(0.025,.645)
46-70	57(60)	182(63.2)	-3.2(-7.7 to 14.6)	.302(.117, .779)
≥71	22(23.2)	39(13.5)	9.7(1.1 to 19.7)	3.31(1.28, 8.55)
Comorbid condition				
No Comorbid	24(25.3)	87(30.2)	-4.9(-5.9 to14.4)	.78(0.46,1.3)
DM	36(37.9)	69(24)	13.9(3.4 to 24.9)	1.9(1.2, 3.1)
CVD	27(28.4)	108(37.5)	-9.1(-2.1to 19)	.66(0.4,1.1)
Old Age	7(7.4)	12(4.2)	3.2(-1.6to10.5)	1.8(0.7,4.8)
Vaccination status				
Vaccinated	9(9.5)	70(18.3)	-8.8(-0.22to-15.4)	.332(0.16,0.69)
Unvaccinated	86(90.5)	218(75.7)	14.8(5.9 to 21.7)	3.01(1.44, 6.25)
BSR mg/dl				
≤200	37(38.9)	195(67.7)	-28.8(-17.2to-39.3)	.147(.076, .285)
201-350	30(31.6)	71(24.7)	6.9(-3.1to17.8)	.321(.158, .651)
351-600	28(29.5)	22(7.6)	21.9(12.9to32)	3.11(1.54, 6.33)
D-dimers ng/ml				
501-5000	28(29.5)	225(78.1)	-48.6(-37.5to-57.9)	0.11(.06, .19)
5000-10,000	39(41)	34(11.8)	29.3(19to39.9)	5.20(3.02, 8.9)
>10,000	19(20)	17(5.9)	14.1(6.5to23.5)	3.9(1.9, 8.04)
Anticoagulation dose and type				
Prophylactic	41(43.2)	81(28.1)	15.1(4.1to26.3)	1.9(1.2, 3.1)
Therapeutic	48(50.5)	206(71.5)	-21(-9.7to-32)	0.41(0.3, 0.6)
Oral anticoagulants	59(62.1)	147(51)	11.1(-0.46to21.8)	1.6(0.97, 2.5)
LMWH	30(31.6)	133(46.2)	-14.6(-3.18to-24.9)	0.53(0.32, 0.9)

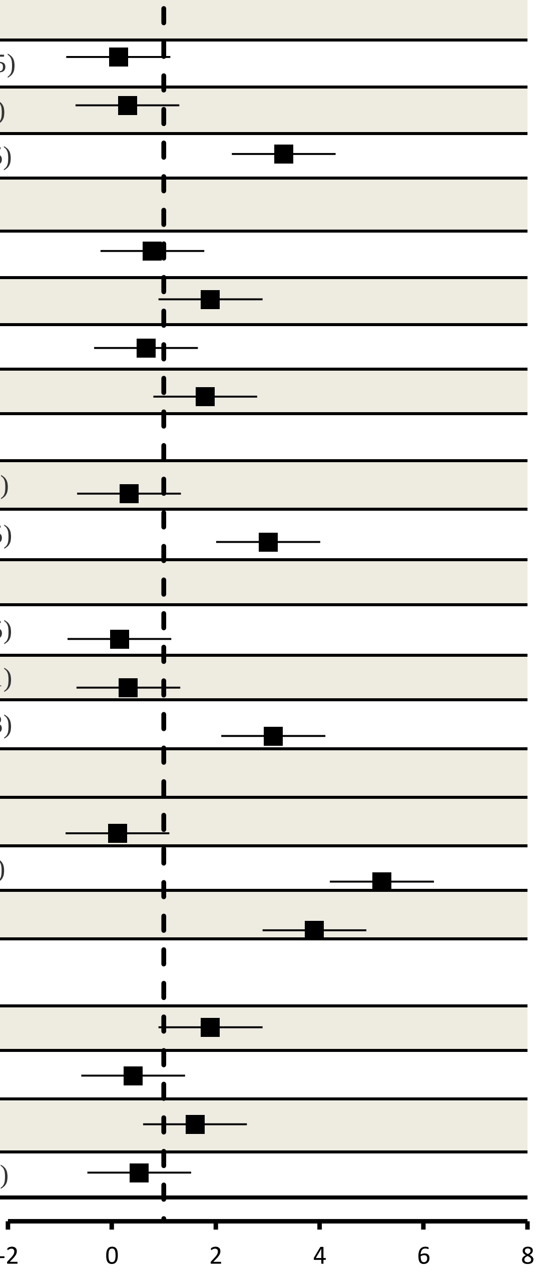

Figure.1. DM (Diabetese Mellitus), CVD(cardiovascular Diseases) , BSR (blood sugar random) , LMWH (Low molecular weight heparin). Association between predictor variables and outcome of neurological complication is measured in term of OR 95% CI shown in forest plot. . In this model, an odds ratio (OR) less than 1.0 showed that neurological complications were associated to a lower risk of exposure, whereas an OR greater than 1 indicated a higher likelihood of exposure. The following equation was used to determine the exposure effect for the prevention or occurrence of neurological complications: exposure predictability of neurological complication= (1- OR) 100%.

Disease progression to death and survival outcome is considered as cases and controls in subgroup analysis of patients with neurological sequelae. The same predictors were investigated for the outcome, OR and p values are given in table.2.

Table 2. Association between predictor variables and death outcome among patients with neurological complications.

Characteristics	Deaths n(%)	Survivals n(%)	OR 95%CI	p value
Total no.	82	13		
Age yrs, Median IQR	59(30,110)	46(27,67)		
Age group				
25-45 yrs.	10(12.2)	6(46.2)	.162(.045 to .58)	<.01
46-70 yrs.	50(61)	7(53.8)	1.34(.412 to 4.35)	>.05
≥71 yrs.	22(26.8)	0	10.04(.57, 176.02)	>.05
No. of comorbid, IQR	1(0,4)	0(0,3)		
No risk factor	16(19.5)	8(61.5)	.151(.044, .525)	.003
DM	34(41.5)	2(15.4)	3.895(.811, 18.72)	.09
CVD	24(29.3)	3(23.1)	1.379(.349, 5.46)	.65
Pulmonary disease	1(1.2)	0	.496(.019,12.84)	.7
Old Age	7(8.5)	0	2.68(.144, 49.77)	.51
Vaccination Status				
Vaccinated	5(6.1)	4(30.8)	.146(.033, .64)	<.05
Unvaccinated	77(93.9)	9(69.2)	6.84(1.55, 30.22)	<.05
Neurological manifestation				
Cerebrovascular Accidents	63(76.8)	4(30.8)	7.46(2.06, 26.96)	<.01
GBS	2(2.4)	5(38.4)	.04(.007, 0.24)	<.001
Encephalitis	17(20.7)	4(30.8)	.588(.16, 2.14)	.422
D-dimers, median(IQR)	6500(280,13000)	1500(350,7800)		
≤500	5(6.1)	4(30.8)	.144(.033, .63)	.016
501-5000	21(25.6)	7(53.8)	.29(.09,.97)	.04
5000-10,000	37(45.1)	2(15.4)	4.52(.94, 21.69)	.05
>10,000	19(23.2)	0	8.29(.47, 145.94)	.14
BSR, IQR	232(80,578)	167(120,429)		
<200	30(36.6)	7(53.8)	.16(.02, .137)	.095
201-350	25(30.5)	5(38.5)	.185(.02, 1.69)	.136
351-600	27(32.9)	1(7.7)	5.40(.590, 49.46)	.136
Anticoagulation dose				
No anticoagulant	6(7.3)	0(0)		
Prophylactic	34(41.5)	7(53.8)	.620(.186, 2.06)	>.05
Therapeutic	42(51.2)	6(46.2)	1.612(.484,5.37)	>.05
Hypoxemia at Admission	75(91.5)	2(15.4)	58.92(10.83, 320.67)	<.001

Figure 2.

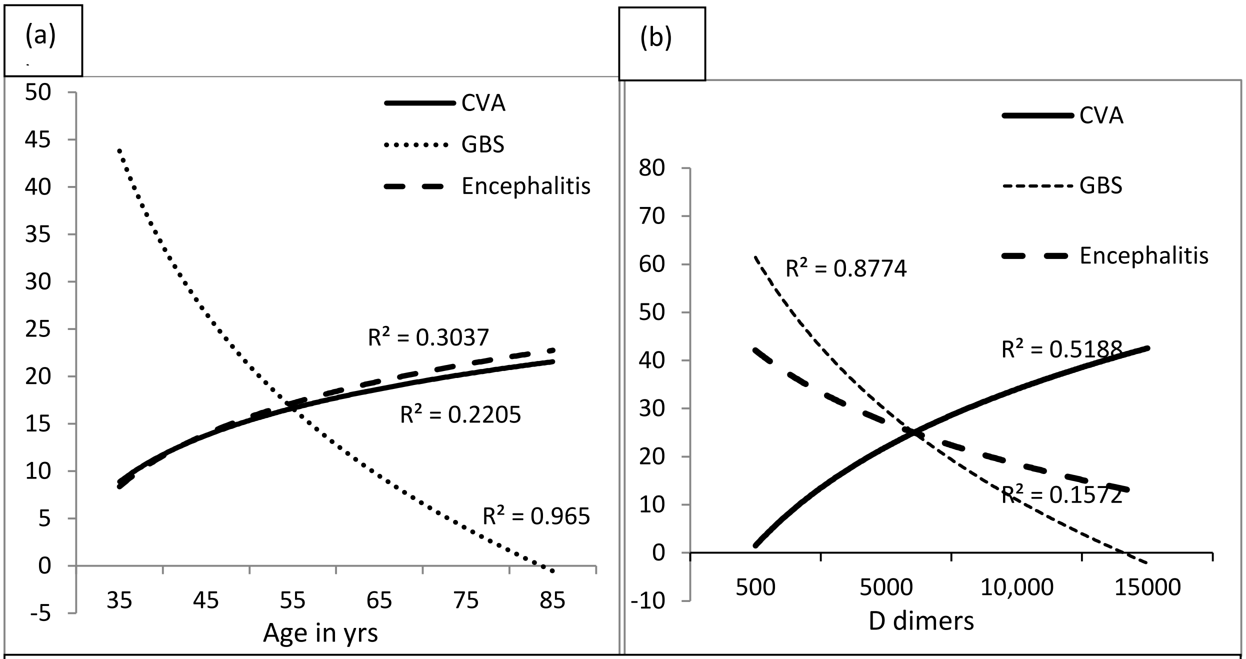

Figure.2 CVA (Cerebrovascular Accidents), GBS (Guillain Barre Syndrome). (a) Shows patients with GBS were from young age group, there was decline in cases with increase in age, Cerebrovascular Accidents were more in old age group and same with encepahlaitis.(b) shows relation between D-dimers and neurological manifestation, patients with GBS had negative correlation with D-dimers whereas patients with CVA had positive correlation elaborated as R^2 value.

Discussions

In this analysis of adults hospitalized for COVID-19 between mid-July 2021 to mid-August 2021(delta period in Pakistan), old age, presence of Diabetes Mellitus and Cardiovascular diseases as comorbid factor, unvaccinated status, raised blood sugar and D-dimer level at admission, severe disease, presence of hypoxemia at admission and prophylactic dose anticoagulation were found to be risk factors for developing neurological complication. Only young age and completely vaccinated status were revealed to be protective factors against death outcome in a subgroup analysis of people with neurological complications. Rest of the factors, on the other hand, had no statistical significance.

aOR in this analysis corresponds to increased risk of neurological complications in older age group of COVID-19 patients.(1)(13) Whereas in a previous study young age group is found to be risk factor for neurological complications. But the majority neurological manifestations were

nonspecific in the study. (14) Those with Diabetes Mellitus as a comorbid factor with severe covid-19 were more likely to develop neurological sequelae, whereas patients with cardiovascular illness had no such risk.(11)

Vaccination was less likely among participants with neurological complication. Estimated vaccine effectiveness against serious neurological sequelae among COVID-19 breakthrough infections is 66.9%, which is comparable to vaccine effectiveness against prevention of hospitalizations and disease progression to death among COVID-19 admitted patients for respiratory complications.(15)

Patients with severe hyperglycemia (>350 mg/dl) were 3.1 times more likely to suffer neurological complications, while patients with markedly raised D-dimers (> 5,000ng/µl) were 5.2 times more probable. Patients on prophylactic dose anticoagulation were 1.9 times more likely to develop neurological complications, while those on therapeutic dose anticoagulation had 59 % lower chances of developing the sequelae. (16)(17) while there was no significant effect of therapeutic anticoagulation on death outcome among patients who already developed neurological complications.(18) In comparison to oral anticoagulation those participants who were on LMWH (enoxaparin) had 47% lesser chances of getting the neurological complication.

In COVID-19 patients who have neurological complications, the risk of disease progression to death is 7.6 times higher than in those who do not.(11) Almost 80% patients had severe respiratory disease at time of hospitalization. So, patients with severe COVID-19 are more likely to get neurological complications(19).

Among patients who developed neurological complications Cerebrovascular events (76.8%) were the most common presentation, followed by encephalitis and GBS.(9)(20) In this group, 18.9% of patients did not have any hypoxemia at the time of admission to the hospital, while 20% of patients had moderate respiratory complications, highlighting that neurological complications can occur as distinct and substantial consequence of COVID-19. When analyzing the risk factors for mortality and survival among patients with neurological complications, research indicates that young patients with no risk factors, who are fully vaccinated, have a low D-dimer, and do not have hypoxemia at the time of hospitalization are more likely to survive and have a better outcome. There was no significant association between the rest of the risk factors evaluated and

death or survival outcomes in these patients. Mostly, 71.4% patients with GBS had good prognosis.(21) Whereas, there was great mortality among other type of complications i.e. Stroke and Encephalitis.(14)(6)

Conclusion

Old age, diabetes mellitus as a comorbid factor, unvaccinated status, high BSR and D-dimers at admission, and prophylactic dosage anticoagulation were all determined to be significant risk factors for neurological complications. However, they have no significant role in predicting the mortality among this group. The most common and fatal neurological complication was found to be cerebrovascular accidents, followed by encephalitis and GBS. Because patients who developed neurological complications had an unexpectedly high mortality rate, this COVID-19 complication should not be overlooked among admitted patients, and each patient should be thoroughly examined for any neurological manifestations to prioritize them and consider any preventable consequences.

Limitations of study

Retrospective nature of study and different types of neurological complications among cases are limitations of study. We need longitudinal prospective studies among COVID-19 patients in order to establish strength to observations. Moreover, study duration was short and only included delta period so the result cannot be generalized to all variants of SARS-COV-2.

Conflict of Interest

Authors declare no conflict of interest. There is no funding for this study.

References

1. Romero-Sánchez CM, Díaz-Maroto I, Fernández-Díaz E, Sánchez-Larsen Á, Layos-Romero A, García-García J, et al. Neurologic manifestations in hospitalized patients with COVID-19: The ALBACOVID registry. Neurology. 2020;95(8):e1060–70.

2. Pantelis C, Jayaram M, Hannan AJ, Wesselingh R, Nithianantharajah J, Wannan CMJ, et al. Neurological, neuropsychiatric and neurodevelopmental complications of COVID-19. Aust N Z J Psychiatry. 2021;55(8):750–62.

3. Pryce-Roberts A, Talaei M, Robertson NP. Neurological complications of COVID-19: a preliminary review. J Neurol. 2020;267(6):1870–3.

4. Brucki SMD, Corazza LA, De Queiroz AP, Barros MP, Tatsch JFS, Riso IL, et al. Neurological complications in COVID-19 patients from Latin America. Brain.

2021;144(3).

5. Moghaddar M, Radman R, Macreadie I. Severity, pathogenicity and transmissibility of delta and lambda variants of SARS-CoV-2, toxicity of spike protein and possibilities for future prevention of COVID-19. Microorganisms. 2021;9(10).

6. Morassi M, Bagatto D, Cobelli M, D'Agostini S, Gigli GL, Bnà C, et al. Stroke in patients with SARS-CoV-2 infection: case series. J Neurol. 2020;267(8):2185–92.

7. Al-Ramadan A, Rabab'h O, Shah J, Gharaibeh A. Acute and post-acute neurological complications of COVID-19. Neurol Int. 2021;13(1):102–19.

8. Shehata GA, Lord KC, Grudzinski MC, Elsayed M, Abdelnaby R, Elshabrawy HA. Neurological complications of covid-19: Underlying mechanisms and management. Int J Mol Sci. 2021;22(8).

9. Orrù G, Conversano C, Malloggi E, Francesconi F, Ciacchini R, Gemignani A. Neurological complications of covid-19 and possible neuroinvasion pathways: A systematic review. Int J Environ Res Public Health. 2020;17(18):1–18.

10. Almutairi MM, Sivandzade F, Albekairi TH, Alqahtani F, Cucullo L. Neuroinflammation and Its Impact on the Pathogenesis of COVID-19. Front Med. 2021;8(November):1–14.

11. Nannoni S, de Groot R, Bell S, Markus HS. Stroke in COVID-19: A systematic review and meta-analysis. Int J Stroke. 2021;16(2):137–49.

12. Sundaramurthy R, Balasubramanian S, Ganesan V, Aggarwal P, Parvataneni T, Jyothi Ramachandran Nair DP, et al. Clinical and Laboratory Factors in Predicting Mortality Among COVID-19 RT-PCR Positive Patients: A Retrospective Observational Study From a Tertiary Care Center. Cureus. 2021;13(11).

13. Meppiel E, Peiffer-smadja N, Maury A, Bekri I, Desestret V, Gorza L, et al. Neurological manifestations associated with SARS-CoV-2 and other coronaviruses: A narrative review for clinicians. 2020;(January). https://doi.org/10.1016/j.neurol.2020.10.001

14. Liotta EM, Batra A, Clark JR, Shlobin NA, Hoffman SC, Orban ZS, et al. Frequent neurologic manifestations and encephalopathy-associated morbidity in Covid-19 patients. Ann Clin Transl Neurol. 2020;7(11):2221–30.

15. Tenforde MW, Self WH, Adams K, Gaglani M, Ginde AA, McNeal T, et al. Association Between mRNA Vaccination and COVID-19 Hospitalization and Disease Severity. Jama. 2021;30329.

16. Therapeutic Anticoagulation with Heparin in Critically Ill Patients with Covid-19. N Engl J Med. 2021;385(9):777–89.

17. Connors JM, Brooks MM, Sciurba FC, Krishnan JA, Bledsoe JR, Kindzelski A, et al. Effect of Antithrombotic Therapy on Clinical Outcomes in Outpatients with Clinically Stable Symptomatic COVID-19: The ACTIV-4B Randomized Clinical Trial. JAMA - J Am Med Assoc. 2021;326(17):1703–12.

18. Sholzberg M, Tang GH, Rahhal H, Alhamzah M, Kreuziger LB, Áinle FN, et al. Effectiveness of therapeutic heparin versus prophylactic heparin on death, mechanical ventilation, or intensive care unit admission in moderately ill patients with covid-19 admitted to hospital: RAPID randomised clinical trial. BMJ. 2021;375.

19. Harapan BN, Yoo HJ. Neurological symptoms, manifestations, and complications associated with severe acute respiratory syndrome coronavirus 2 (SARS-CoV-2) and coronavirus disease 19 (COVID-19). J Neurol. 2021;268(9):3059–71.

20. Merkler AE, Parikh NS, Mir S, Gupta A, Kamel H, Lin E, et al. Risk of Ischemic Stroke in Patients with Coronavirus Disease 2019 (COVID-19) vs Patients with Influenza. JAMA Neurol. 2020;77(11):1366–72.

21. Abu-Rumeileh S, Abdelhak A, Foschi M, Tumani H, Otto M. Guillain–Barré syndrome spectrum associated with COVID-19: an up-to-date systematic review of 73 cases. Vol. 268, Journal of Neurology. Springer Berlin Heidelberg; 2021. 1133–1170 p.

Figure Click here to access/download;Figure;Figures.docx

Figure 1. Association between predictor variables and development of neurological complications

Characteristics	Cases n (%)	Controls n (%)	Absolute difference 95%CI	Adjusted OR 95%CI
Age groups, yrs				
25-45	16(16.8)	67(23.3)	-6.5(-3.4 to 14.6)	.126(0.025,.645)
46-70	57(60)	182(63.2)	-3.2(-7.7 to 14.6)	.302(.117, .779)
≥71	22(23.2)	39(13.5)	9.7(1.1 to 19.7)	3.31(1.28, 8.55)
Comorbid condition				
No Comorbid	24(25.3)	87(30.2)	-4.9(-5.9 to14.4)	.78(0.46,1.3)
DM	36(37.9)	69(24)	13.9(3.4 to 24.9)	1.9(1.2, 3.1)
CVD	27(28.4)	108(37.5)	-9.1(-2.1to 19)	.66(0.4,1.1)
Old Age	7(7.4)	12(4.2)	3.2(-1.6to10.5)	1.8(0.7,4.8)
Vaccination status				
Vaccinated	9(9.5)	70(18.3)	-8.8(-0.22to-15.4)	.332(0.16,0.69)
Unvaccinated	86(90.5)	218(75.7)	14.8(5.9 to 21.7)	3.01(1.44, 6.25)
BSR mg/dl				
≤200	37(38.9)	195(67.7)	-28.8(-17.2to-39.3)	.147(.076, .285)
201-350	30(31.6)	71(24.7)	6.9(-3.1to17.8)	.321(.158, .651)
351-600	28(29.5)	22(7.6)	21.9(12.9to32)	3.11(1.54, 6.33)
D-dimers ng/ml				
501-5000	28(29.5)	225(78.1)	-48.6(-37.5to-57.9)	0.11(.06, .19)
5000-10,000	39(41)	34(11.8)	29.3(19to39.9)	5.20(3.02, 8.9)
>10,000	19(20)	17(5.9)	14.1(6.5to23.5)	3.9(1.9, 8.04)
Anticoagulation dose and type				
Prophylactic	41(43.2)	81(28.1)	15.1(4.1to26.3)	1.9(1.2, 3.1)
Therapeutic	48(50.5)	206(71.5)	-21(-9.7to-32)	0.41(0.3, 0.6)
Oral anticoagulants	59(62.1)	147(51)	11.1(-0.46to21.8)	1.6(0.97, 2.5)
LMWH	30(31.6)	133(46.2)	-14.6(-3.18to-24.9)	0.53(0.32, 0.9)

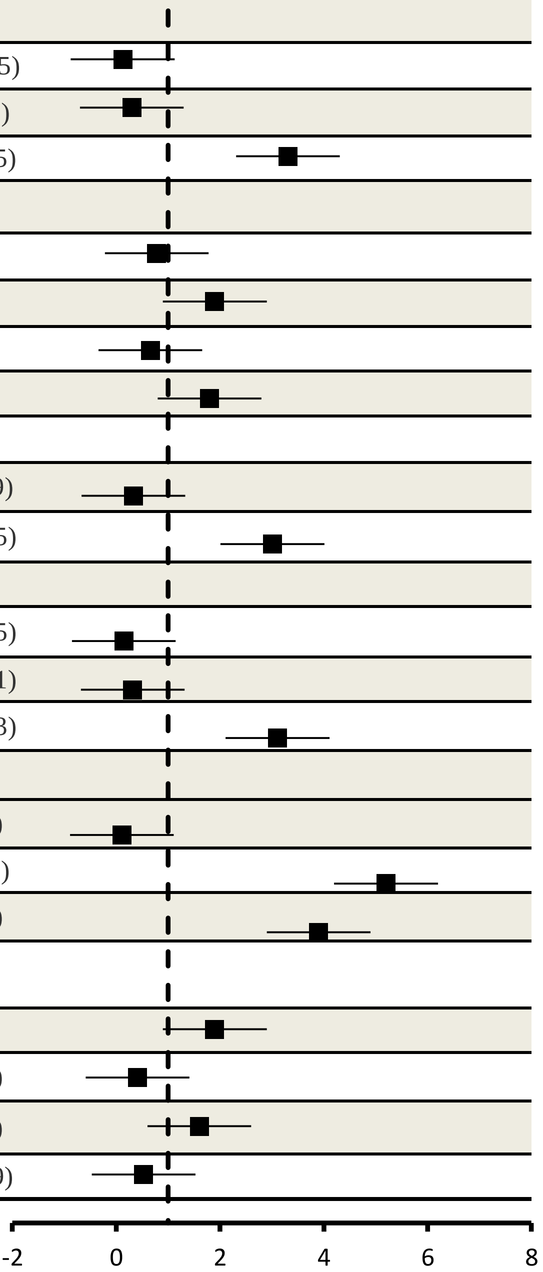

Figure.1. DM (Diabetese Mellitus), CVD(cardiovascular Diseases) , BSR (blood sugar random) , LMWH (Low molecular weight heparin). Association between predictor variables and outcome of neurological complication is measured in term of OR 95% CI shown in forest plot. . In this model, an odds ratio (OR) less than 1.0 showed that neurological complications were associated to a lower risk of exposure, whereas an OR greater than 1 indicated a higher likelihood of exposure. The following equation was used to determine the exposure effect for the prevention or occurrence of neurological complications: exposure predictability of neurological complication= (1- OR) 100%.

Figure 2.

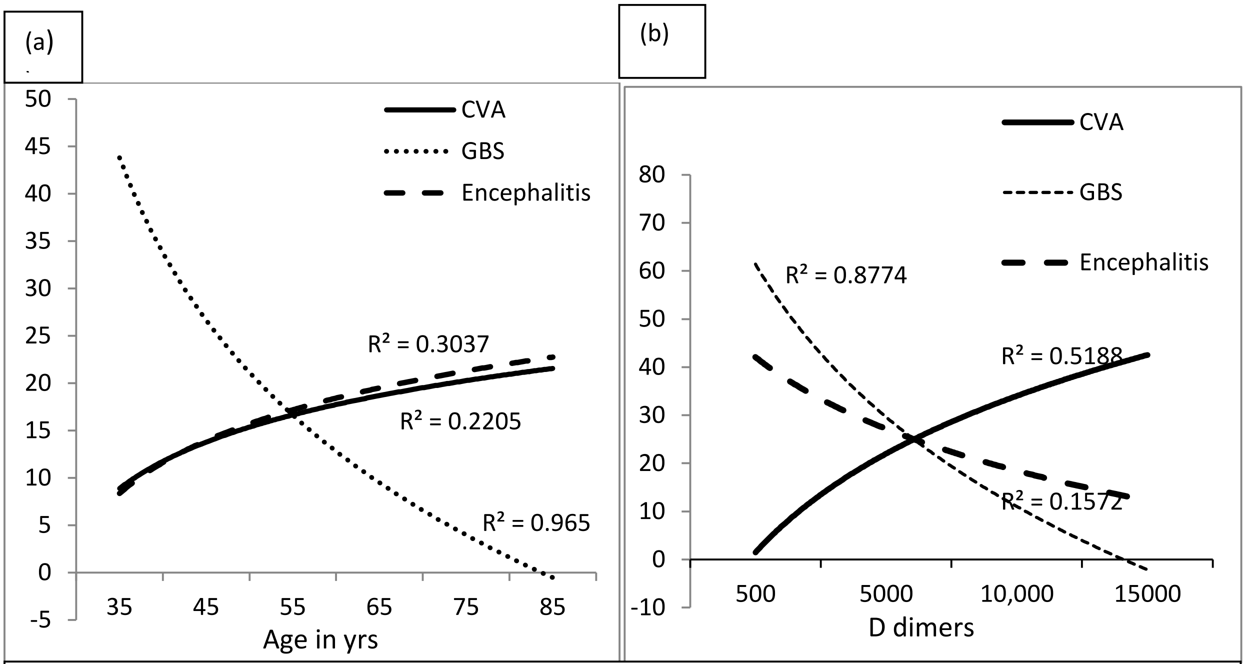

Figure.2 CVA (Cerebrovascular Accidents), GBS (Guillain Barre Syndrome). (a) Shows patients with GBS were from young age group, there was decline in cases with increase in age, and Cerebrovascular Accidents were more in old age group and same with Encepahlaitis. (b) Shows relation between D-dimers and neurological manifestation, patients with GBS had negative correlation with D-dimers whereas patients with CVA had positive correlation elaborated as R^2 value.
