# Supplementary material for: Predictors of fatal neurological complications among admitted COVID-19 patients with their implication in outcome: A Case Control study
Source: PLoS One. 2022 Sep 26;17(9):e0274485. doi: 10.1371/journal.pone.0274485 (PMC9512176; doi:10.1371/journal.pone.0274485)
Supplement: Attachment — Submitted filename: Response to Reviewers.docx [file pone.0274485.s003.docx]

Soham Bandyopadhyay
Academic Editor
PLOS ONE	Dated: 29/07/2022s

Dear Editor,

Re: Resubmission of manuscript after revision: PONE-D-22-05696

Thank you for the email enclosing the reviewer’s response on the manuscript. Please find attached a revised version of our manuscript entitled,” Predictors of Fatal Neurological Complications among Admitted COVID-19 Patients with their Implication in Outcome: A Case Control Study” We have carefully reviewed the comments and revised the manuscript accordingly. Our response are given point by point to reviewer’s comments. Revisions are highlighted in red color within the manuscript. We hope that revised manuscript is now suitable for publication and looking forward to your response.

Yours’ sincerely

Dr Javaria Aslam

Assistant Professor Medicine

Corresponding author

**Academic Editors:**

1. Please ensure that your manuscript meets PLOS ONE's style requirements, including those for file naming. The PLOS ONE style templates can be found at 
https://journals.plos.org/plosone/s/file?id=wjVg/PLOSOne_formatting_sample_main_body.pdf and 
https://journals.plos.org/plosone/s/file?id=ba62/PLOSOne_formatting_sample_title_authors_affiliations.pdf

2. Please amend your current ethics statement to address the following concerns:
a) Did participants provide their written or verbal informed consent to participate in this study?
b) If consent was verbal, please explain i) why written consent was not obtained, ii) how you documented participant consent, and iii) whether the ethics committees/IRB approved this consent procedure.

3. We note that you have stated that you will provide repository information for your data at acceptance. Should your manuscript be accepted for publication, we will hold it until you provide the relevant accession numbers or DOIs necessary to access your data. If you wish to make changes to your Data Availability statement, please describe these changes in your cover letter and we will update your Data Availability statement to reflect the information you provide.

4. Please include your full ethics statement in the ‘Methods’ section of your manuscript file. In your statement, please include the full name of the IRB or ethics committee who approved or waived your study, as well as whether or not you obtained informed written or verbal consent. If consent was waived for your study, please include this information in your statement as well.

5. Please remove your figures from within your manuscript file, leaving only the individual TIFF/EPS image files, uploaded separately.  These will be automatically included in the reviewers’ PDF.

**Response to Academic editor:**

We much appreciate your consideration of this paper for publication. We have meticulously rectified the shortcomings identified by the academic editor.

1. The manuscript has been modified to adhere to the journal's specifications.
2. The revised manuscript includes a clear statement that written consent was obtained from study participants.
3. Dataset has been given in supporting information in xlsx file.
4. Full ethic statement is included in the methodology section of revised manuscript.
5. Figures are withdrawn from the manuscript file and converted to TIFF format in separate files.

**Reviewer #1:**

1. Keywords should include important terms like Cerebrovascular events, and COVID 19. The keywords used are general, non-specific, and should be edited.
2. When mentioning reference numbers in the parenthesis, a full stop should be following the references at end of the sentence, not preceding the references.
3. Line 78-82: Is the author trying to suggest an increased risk of neurological disease with Delta variant as compared to COVID 19 in general? Please clarify for the readers.
4. Please abstain from a colloquial style of wiring that will also remove redundancy from the text.
5. Instead of mentioning molecular testing for COVID, please mention the detail with the type of PCR and instrumentation used for testing.
6. Line 150 - Were partially vaccinated patients included or excluded from the study? It should be clarified.
7. Line 192 - 18 patients among the cases had no hypoxemia at the time of admission, please state what was the reason for admission. Neurological symptoms or any other criteria used for admission should be mentioned.
8. Line 197-198 It will be interesting to mention respiratory illness severity and type of respiratory support in relation to neurological complications as increased risk of dying from covid 19.

**Response to Reviewer #1:**

Thank you very much for your valuable insight into our work. We have addressed your comments carefully

1. In the revised manuscript, Key words have been edited and important terms such as cerebrovascular events, COVID-19, encephalitis, predictors, and outcome have been added.
2. Full stops are correctly placed following the reference number at the end of sentences.
3. The study was conducted during the delta period of SARS-CoV-2 in Pakistan, which was the worst in terms of disease spread, severity, and fatality. Moreover, more neurological problems were reported compared to earlier waves. Therefore, it was important to highlight its fatality in the introduction, despite the fact that it was not compared to the other waves and, as stated in the study's limitations, its results cannot be generalized.
4. Informal writing style has been avoided as much as possible in the revised version.
5. Type and specifications of SARS-COV-2 PCR testing have been described in detail in the revised manuscript's methodology section.
6. Only fully vaccinated or unvaccinated participants were included in the study, excluding partially vaccinated patients from both cases and controls. A clear statement on vaccination status is included in both the methodology and results sections of revised manuscript.
7. The research was conducted at a hospital that received COVID-19 confirmed cases from other tertiary care hospitals. In the setting of a pandemic, the hospital received all sick admissions with positive PCR or HRCT findings diagnostic of COVID pneumonia with acute onset symptoms. Eighteen patients did not have hypoxemia at the time of hospitalization, but had positive PCR for SARS-COV-2 and extra pulmonary symptoms such as neurological or HRCT findings suggestive of COVID pneumonia without hypoxemia.
8. All patients with neurological complications were evaluated for the severity of respiratory illness, termed "Disease severity," which was measured by the WHO COVID-19 clinical progression scale in the methodology section. According to the scale, patients with moderate disease had no hypoxemia and did not require any oxygen support, patients with severe disease had hypoxemia and required oxygen support via simple face mask or rebreathing mask, and patients with critical illness required noninvasive or invasive ventilator support and required ICU care in general.

**Reviewer #2:** This article requires an overhaul to address major concerns with formatting, stylistic approaches, grammar, methodology, and the presentation of your findings. While significant as a study from this corner of the world, it requires a more scientific approach towards your statistical inclusion of predictors, case-matching strategy, and extrapolation of findings. I believe with improvements and revisiting your data, this study can become a significant and worthy publication.

**Response to Reviewer #2:**

Thank you very much for your valuable comments for our original work. We have carefully put an effort to revise all sections of the manuscript to satisfy the reviewer's major concerns. Errors in grammar and sentence structure have been addressed thoroughly. Red highlighting denotes alterations to the manuscript.

Abstract:

Background in the abstract section has been edited for grammatical errors and extended to make it more comprehensive. Result section of structured abstract has been revised, clearly explaining the OR in scientific way. Conclusion of abstract also revised to conclude the results in a meaningful and scientific way.

Introduction:

Highlighted sentence has been edited with required changes and two more refernces on # 13 and 14 are added.

Methadology:

The methodology section has been thoroughly revised while preserving the original methodology. In the first paragraph of the methodology, the duration, design, and location of the study, as well as the ethical statement and design principles, are outlined in detail. The following paragraph describes the participants who defined the cases and matched controls. In the following paragraphs, predictor variables and primary and secondary outcome measures are discussed in detail in primary and subgroup analysis. In the last statistical analysis is mentioned. In order to make the methodology section more comprehensible for the readers, the majority of the text is rearranged and modified with pertinent amendments.

1. Division name is mentioned as Bahawalpur, Punjab, Pakistan.
2. Mild and serious neurological complications are described with example in the section where cases, controls and, inclusion and exclusion criteria are described.
3. Hospital admission criteria was positive PCR or HRCT chest suggestive of COVID-19 pneumonia and its related complications e.g high grade persistent fever, hypoxemia needing respiratory assistance, serious neurological complications , cardiac complications like myocarditis or renal complications like acute kidney injury.
4. In order to avoid lead time bias, controls are recruited within two weeks after case enrollment. Since COVID-19 presentation is with a brief history of febrile illness and pulmonary and extra pulmonary complications, it has an abrupt onset. While defining the potential study participants, it was stated that they must be admitted within 10 days after symptom onset or a positive PCR result. Obtaining a patient's PCR testing history was a second method for excluding participants who had persistent positive test. Covid-19.pshealthpunjab.gov.pk, provides a record of each positive or negative test conducted on the individual based on national Identity number. Patients with long history of symptom onset or positive PCR test, for more than 10 days were excluded.

Results:

The results section has been revised by revisiting the data and rearranging the results. With the aid of text, table 1 and figure 1, the main analysis’ results are outlined in detail. Afterwards, a case-only (subgroup) analysis is presented using text and table 2. In the last section, different neurological manifestations were evaluated independently in relation to predictor variables and outcome variables. To display the results of this investigation in a scientific manner, fig. 2 and a new table 3. Were incorporated.

While revisiting and presenting the data in a more effective manner, its originality was kept intact and no changes were made to the original data or statistics.

The discussion section has been meticulously revised to eliminate all grammatical mistakes. The first paragraph is substantially modified by incorporating additional results' conclusions.

The conclusion section is rewritten to better summarize the study's findings and provide valuable suggestions.
